# The Treatment and Management of Asthma1An abstract of a lecture delivered to the class in the Philadelphia Polyclinic, November, 1892.

**Published:** 1892-12

**Authors:** Thomas J. Mays

**Affiliations:** Professor of Diseases of the Chest in the Philadelphia Polyclinic, and Visiting Physician to the Rush Hospital for Consumption, of Philadelphia


### THE TREATMENT AND MANAGEMENT OF ASTHMA.[1]

By THOMAS J. MAYS, M. D.,

Professor of Diseases of the Chest in the Philadelphia Polyclinic, and Visiting Physician to the Rush Hospital for Consumption, of Philadelphia.

ASTHMA is a paroxysmal disease of the pneumogastric nerves, which throws the muscular fibers of the bronchial tubes into spasmodic contraction. Its prominent symptoms are itching of the head and neck, oppression and tightness of the chest, dyspnea, bloating of the abdomen, pain in the region of the diaphragm, cough, expectoration, and fever. Its causes are predisposing and exciting. (1) It may be inherited as asthma, and it may appear in children who come from consumptive or nervous families. It seems as if there is a predisposition necessary before the disease can develop. (2) Among the exciting causes are the inhalation of dust, powdered ipecacuanha, pollen of grasses and of roses, odors of certain animals, as cats, sheep, etc. Reflex excitation coming from the nose, stomach, liver, intestines, uterus, etc. Its relation to hay fever is very close. Practically, there is no difference between the two. I find that that which relieves the one will also relieve the other.

Its treatment resolves itself into that (1) which aims to give immediate relief from the paroxysm, and (2) that which aims to prevent a recurrence of the paroxysm. Those remedies which relieve the paroxysm may be classified as follows: (1) Central narcotics, consisting of morphine, belladonna, strammonium hyoscyamus, tobacco, chloroform, ether, ethyl bromide, ipecacuanha, sangunaria, etc., and (2) the peripheral narcotics—amyl nitrite, sodium nitrite, pilocarpine, etc. Now, all our more or less powerful therapeutic agents are stimulants to the general or special bodily tissues which they affect, in small doses; while in large doses they paralyze the same. All the above-named agents only relieve asthma when given in large or paralyzing doses: the central narcotics exerting their influence on the central nervous system; the emetics acting on the pneumogastric filaments, while the

---

1. An abstract of a lecture delivered to the class in the Philadelphia Polyclinic, November, 1892.

peripheral narcotics paralyze the vaso-motor or sympathetic nerves, which supply the unstriped muscular fibers of the bronchial mucous membrane and blood-vessels. While all these agents relieve asthma, and, indeed, in some cases are indispensable, it is quite clear that, in doing so, they lower or depress the functions of the parts on which they act, and that they do not, therefore, come up to the ideal of an asthmatic remedy. The best among them are nitro-glycerine, one or two minims of a one-per-cent. solution every three or four hours by the mouth, and one-twentieth or one-tenth of a grain of morphine, hypodermically, once or twice a day.

What, then, is the remedy which may be given continuously for the alleviation of this disease, and without the undesirable effects of the above-named classes? Which drug will relieve asthma in stimulant doses? Such a drug, I believe, we possess in strychnine. Of course, we must bear in mind that all stimulants are only supplementary agents which maintain the functions of the body without adding material support to the same; but there is also good reason for believing that they cause the tissues to appropriate a larger amount of nutritive material than they would otherwise do, and in this way our stimulant drugs become tissue builders. It has been shown that the power of strychnine in this respect is greater than that of any other stimulant. This drug has a special affinity for the nervous system, which action is especially accentuated on the respiratory center and pneumogastric nerves. In stimulant doses it gives a supporting influence to the respiratory movements, and, unlike morphine, lobelia, belladonna, or nitro-glycerine, it does not depress or narcotize the nervous system.

Asthma being a spasmodic disease, in what manner does strychnine bring relief? How does it act as antispasmodic? The most probable theory of the spasmodic state is, that there is at the beginning of the paroxysm a superabundant discharge of nerve force through the pneumogastric nerves, which throws the bronchial muscles into contraction. But, whatever the intimate nature of this condition may be, it is evidence of degradation or nerve weakness; and strychnine, by elevating the tone of these nerves, increases the controlling power of the same. A stimulant dose of strychnine will depend on the age of the patient, and the length of time during which the drug has been given, although asthmatics, as a rule, will bear larger doses of strychnine than most other patients. Begin, as a rule, with one-thirtieth of a grain subcutaneously once a day, and gradually increase to one-twentieth or one-tenth of a grain, or more if necessary to impress the system with its full stimulant effects. Do not waste your time with small doses. To these amounts of strychnine small doses of from one-four-hundredth to one-six-hundredth of a grain of atropine may be added. It is best to administer these drugs in the evening, because asthma is nocturnal in its attacks, and your patient should be protected at night, so he can sleep. Additionally to its hypodermic use, this drug may be given in the following combination:

℞. Phenacetini.............................gr. lxiv.
Quiniæ Sulph.............................gr. xxxii.
Ammon Murias............................ ʒiss.
Pulv. Capsici............................ gr. iv.
Strychninæ Sulph........................ gr· 1⅛.
M. Ft. Capsulas No. xxxii.
Sig.: One capsule four times a day.

Or in the following:

℞. Strychnine Sulph........................ gr. 1⅛
Syr. Acid Hydriodici..................... ℥ij.
Syr. Hypophosph......................... aa fl.
M. Sig.: One teaspoonful four times daily.

In fact, light cases of asthma require no hypodermic injection, and do well enough when the above-named preparations are given. In severe cases it is, of course, advisable to add morphine or nitroglycerine to the strychnine and atropine treatment, especially at the beginning. This treatment will break up the paroxysms, but even after they are broken, many old asthmatics still remain in the most abject misery. They may be compelled to sit up day and night panting for breath, and still labor under the impression that they are suffering from asthma. This is a mistake; it is not asthma, but the natural state of exhaustion which follows asthma. The respiratory movements, as well as the whole nervous system, are almost completely paralyzed. It is the disorder and chaos following the flood. The dyspnea is not paroxysmal as before, but is felt now on the slightest exertion. This stage of the disease is most important from a therapeutic standpoint. Nitroglycerine, lobelia, and other narcotics are of no use. Rest is most essential now. They must do absolutely nothing. Lie down if they can, or sit still. They should even be fed. I have known patients who were breathing comfortably bring on a most severe exhaustion,—dyspnea—by merely undertaking to write a letter. During the rest-treatment give food of the most nourishing character, such as freshly expressed beef juice—a cupful a day—beef-powder, beef, mutton, milk, oysters, clams, etc. In this stage, strychnine is also of the greatest value. Massaging is also to be used in desperate cases. Electricity is also of great service. So are rarefied air and calisthenic exercises obtained in the pneumatic cabinet treatment. To procure sleep at night, morphine may be added to the hypodermic injections of strychnine.

Success in treating asthma depends as much on the proper management of the individual as it does on the administration of drugs in the proper doses and at the proper time. Principles can only be carried out by paying attention to details; hence, each patient must be under the complete control of his physician in regard to his food, medicines, exercise, and everything else. This pertains particularly to old asthmatics who are constant sufferers. If the instruction which is given this evening is closely followed, there are very few cases which will not yield; and, as an illustration of what may be done in desperate cases, I will conclude by relating the condensed histories of the two following examples, the second of which is still under occasional observation:

Case I. A., aged 46, a sufferer from asthma for thirty-five years, the attacks becoming more frequent and severe during the last three years. For four weeks before coming under treatment he had been unable to lie down, on account of the disease. The hypodermic injection of strychnine, one twenty-fifth of a grain, and morphine, one-fifteenth of a grain, gave him almost immediate temporary relief. The morphine was discontinued after the second day, and one minim of a one-per-cent. solution of nitro-glycerine every four hours was substituted. The strychnine was gradually increased, and the nitro-glycerine omitted in the course of a week. Additionally, he was kept quiet, received nourishing food, and strychnine by the mouth. In three days he was able to lie down, and in ten days more the asthma had disappeared.

Case II. B., aged 50, an asthmatic for twenty-five years. Daily attacks for one year, during which time he had been unable to lie down, day or night. Came under observation about six weeks ago, and received about the same treatment as the previous case. The relief was prompt after each injection, but the hypodermic injections had to be continued nightly for five weeks to keep the stubborn disease in abeyance. In two weeks he was able to lie down, and is now practically well.

---

A Serious Affliction.—"Well, I see old Mithomer has died at last."

"Yes; it was a sad loss to me."

"I didn't know you were a friend of his."

"No; I was his physician."—*Life.*

